# Survival impact of centralization and clinical guidelines for soft tissue sarcoma (A prospective and exhaustive population-based cohort)

**DOI:** 10.1371/journal.pone.0158406

**Published:** 2017-02-03

**Authors:** Olfa Derbel, Pierre Etienne Heudel, Claire Cropet, Pierre Meeus, Gualter Vaz, Pierre Biron, Philippe Cassier, Anne-Valérie Decouvelaere, Dominique Ranchere-Vince, Olivier Collard, Eric De Laroche, Philippe Thiesse, Fadila Farsi, Dominic Cellier, François-Noel Gilly, Jean-Yves Blay, Isabelle Ray-Coquard

**Affiliations:** 1 Department of Medical Oncology, Centre Léon Bérard, Lyon, France; 2 Department of Biostatistics, Centre Léon Bérard, Lyon, France; 3 Department of Surgery, Centre Léon Bérard, Lyon, France; 4 Groupe Sarcome Français, Groupe d’Etude des Tumeurs Osseuses (GSF-GETO), Lyon France; 5 Department of Orthopedic Surgery, Clinique des IRIS, Lyon, France; 6 Department of Pathology, Centre Léon Bérard, Lyon, France; 7 Institut de Cancérologie Lucien Neuwirth, Saint-Priest en Jarez, France; 8 Department of Radiology, Centre Léon Bérard, Lyon, France; 9 Réseau Espace Santé Cancer, Rhône-Alpes, France; 10 Claude Bernard University, Lyon, France; 11 Laboratoire HESPER EA 7425, Lyon 1 University, Lyon, France; University of Texas MD Anderson Cancer Center, UNITED STATES

## Abstract

**Purpose:**

The outcome of sarcoma has been suggested in retrospective and non-exhaustive studies to be better through management by a multidisciplinary team of experts and adherence to clinical practice guidelines (CPGs). The aim of this prospective and exhaustive population based study was to confirm the impact of adherence to CPGs on survival in patients with localized sarcoma.

**Experimental design:**

Between 2005 and 2007, all evaluable adult patients with a newly diagnosis of localized sarcoma located in Rhone Alpes region (n = 634), including 472 cases of soft-tissue sarcoma (STS), were enrolled. The prognostic impact of adherence to CPGs on progression-free survival (PFS) and overall survival (OS) was assessed by multivariate Cox model in this cohort.

**Results:**

The median age was 61 years (range 16–92). The most common subtypes were liposarcoma (n = 133, 28%), unclassified sarcoma (n = 98, 20.7%) and leiomyosarcoma (n = 69, 14.6%). In the initial management phase, from diagnosis to adjuvant treatment, the adherence to CPGs for patients with localized STS was 36% overall, corresponding to 56%, 85%, 96% and 84% for initial surgery, radiation therapy, chemotherapy and follow-up, respectively. Adherence to CPGs for surgery was the strongest independent prognostic factor of PFS, along with age, gender, grade, and tumor size. For OS, multivariate analysis adherence to CPGs for surgery was a strong independent prognostic factor, with an important interaction with a management in the regional expert centers.

**Conclusions:**

This study demonstrates impact of CPGs and treatment within an expert center on survival for STS patients in a whole population-based cohort.

## Introduction

Clinical practice guidelines (CPGs) provide a set of recommendations for diagnostic and therapeutic procedures, with the aim of improving patient care and disease outcomes [1]. In the field of rare cancer management, development of CPGs is particularly challenging because of the variability of disease presentation and the complexity of therapeutic decisions to interpret scientific data [1]. Thus, to be successfully incorporated into routine practice, CPGs must be evidence-based, extensively validated and well implemented [1,2]. For less frequent types of cancer, difficulties in standardizing and adhering to CPGs are well documented [2].

Sarcomas are a heterogeneous group of connective tissue malignancies that includes more than 50 histological subtypes and more molecular subtypes [3–5]. Soft-tissue sarcoma (STS) have an average annual incidence of 4–5 cases per 100,000 individuals, with an incidence of 1 to 1·5/100000 and 0·6 for 1000000 for gastrointestinal stromal tumor (GIST) and bone sarcoma respectively [5–7]. As for other rare tumors, this rarity implies that most pathologists and physicians have limited experience with the diagnosis and multimodality treatment of STS [8]. Over the last decade, several scientific societies and national groups have developed CPGs dedicated to sarcoma [9,10,11]. However, only a minority of patients is treated according to CPGs in most studies reported so far, specifically during initial management which may impact the most on long-term outcome [12–15].

The influence of CPGs on the management of STS has been explored in several studies, each assessing different parameters [12–17]. Retrospective analyses suggested that optimization of organizational aspects, such as referral procedures, centralization of healthcare management in reference centers and quality control programs, more than CPG’s may lead to substantial improvements in the outcome and survival of patients with sarcomas[16,18]. These studies have also indicated that management in specialized hospitals that see a high volume of rare cancers, with an experienced multidisciplinary team and a sarcoma committee, may have a positive impact on survival outcomes [12,13,15,17]. Due to methodological limitations (in particular due to the selection process, retrospective analysis …), none of these studies has conclusively demonstrated the benefits of CPGs in terms of progression-free survival (PFS) and overall survival (OS) [12–17]. In a previous retrospective study on a sample of patients from the Rhone-Alpes (RA) region of France, we reported that the management of STS was suboptimal, and the first surgical procedure had a significant impact on relapse-free survival [19].

To strongly support this observation with an improved level of evidence compared to all previous publications, a population-based study of all STS patients treated in the RA region was performed between 2005 and 2007. With a median follow up of 62,7 months, this study reported impact of organization, adherence to CPG’s and other prognostic factors on survival of localized STS.

## Methods

### Study design

An exhaustive collection of all new cases of sarcoma in the French RA region was conducted over a two-year period (March 2005 to March 2007) in a prospective manner. Further details of the study design were previously published by Ducimetiere et al [5].

### Data collection

The RA region included during this period a total of 43 pathology laboratories and 158 pathologists. All laboratories in the region consented to participate to the study. Exhaustive patient registration was verified by onsite monitoring visits of all pathology laboratories (S1 Appendix). To confirm diagnosis, tumor biopsies and/or surgical material were reviewed by expert pathologists at a central laboratory [8]. Ethical approval for this study was granted by an independent review board, the French data protection authority (Commission Nationale de l'Informatique et des Libertés or CNIL) in agreement with French laws. There was no human experimentation and no consequences on patient management; therefore, no institutional review board review was required. Following approval by the French ethics committee, all surgeons in the region received an information letter about the study and were asked to inform their patients with sarcoma that their medical records would be reviewed for the study. Patient records and information were anonymized and de-identified prior to analysis.

### Patient selection

**Fig 1:**
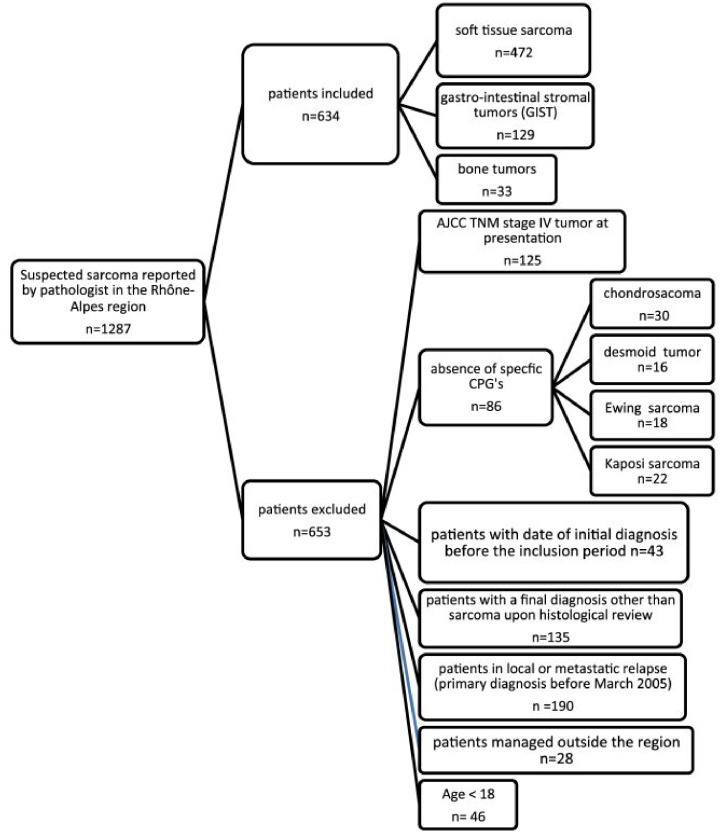
Flowchart of patient selection.

All adult patients with a newly diagnosed primary sarcoma documented by any public or private pathology laboratory in the RA region during the reference period were included in the study. Patient’s selection is detailed in Fig 1. The present study focused only on the data concerning STS (n = 472).

### Clinical practice guidelines

The CPGs used for the present study were the 2004 version of the standards, options and recommendations (SORs) for the clinical management of cancer (http://www.e-cancer.fr/soins/recommandations). These CPGs were developed within a national project coordinated by the French Federation of Comprehensive Cancer Centers as a tool for board-certified oncologists, with no other intended audience.

### Adherence criteria

Adherence criteria and predictive factors for conformity to CPGs were analyzed as reported by Heudel et al [20]. Some of the criteria is detailed below:

**Criteria for optimal initial examination and diagnosis**. Concerns information as size, depth recorded; (ii) computed tomography (CT) or magnetic resonance imaging (MRI) (iii) chest radiograph or CT scan; and (d) pre-operative biopsy (incisional or needle), with some exceptions detailed in the 2004 paper.

**Criteria for optimal histopathology report**. Included (i) diagnosis; (ii) margins; (iii) grade.

Criteria for optimal surgical management included wide excision, negative histological margins (R0). Absence of adjuvant treatment, is acceptable only for superficial, small (<3 cm) and low-grade lesions. Histologically positive margins (R1) or incomplete excision (R2) have to be considered inadequate, and should be followed by further appropriate treatment (further surgery or adjuvant therapy if formal review by a multidisciplinary sarcoma committee considers surgery non-feasible).

**Criteria for optimal radiation therapy management**. For non-operable sarcomas, primary radiation therapy could be an option. Optimal technical criteria are: (i) 50 Gy delivered dose [with an additional boost of 10 Gy in case of microscopic residual tumor (R1)]; (ii) target volume to irradiate encompassing tumor bed, scars, including draining orifices, with adapted security margins; and (iii) delay from surgery to radiation therapy not exceeding 8 weeks.

**Criteria for optimal chemotherapy management**. Non-operable sarcomas, primary chemotherapy can be an option. For operable tumor, neo-adjuvant chemotherapy only as part of a clinical protocol. Adjuvant chemotherapy could be performed for patients with histologically positive margins after wide surgery. Drugs, dose, delay and number of cycles are to be in accordance with CPG’s

Criteria for optimal post-therapeutic follow-up with clinical examination every 12 weeks during the first 3 years, then every 4 months until the fifth year post-management.

### Outcome measures

The main objective was to assess the impact of adherence to CPGs on survival outcomes, including PFS (per investigator evaluation) and OS from diagnosis of primary localized sarcoma to the event.

### Statistical methods

#### Primary endpoint analysis

The impact of adherence to CPGs on survival of STS patients was evaluated in a multivariate analysis including previously validated prognostic factors. Adherence to CPGs was evaluated for the following key steps of patient management: i) diagnosis; ii) primary surgery; iii) adjuvant chemotherapy, iv) radiotherapy, v) follow-up; and vi) global adherence defined as adherence for the whole sequence from diagnosis to post-treatment survey.

PFS was defined as the time from initial diagnosis to disease progression or death from any cause, and censored at the date of last follow-up in the case of no event. OS was defined as the time from initial diagnosis to either death or last follow-up visit. Distributions of PFS and OS were estimated using the Kaplan-Meier method.

#### Sample size

Based on the estimated incidence rate and estimated median progression survival published in the literature, the number of observed events (progressions or deaths when evaluating PFS, and deaths only when evaluating OS) in this studied cohort was consistent with the recommendations reported in the literature; indeed, as recommended by Harrell et al, a minimum of 10 events per covariable included in the model is appropriate for predictive discrimination [21]. Of note though, no specific methodology has been developed for power and sample size calculation in such multivariate analysis in the context of a population-based patient cohort.

#### Analysis of prognostic factors for survival

A total of 11 covariates were analyzed as potential prognostic factors for the survival of STS patients. According to Harrell et al. (see above), sample size determination thus translates in a minimum of 110 outcome events required to have a good accuracy and fitting of the model, and was achieved with the sample size analyzed.

To evaluate the relationship between survival and adherence to CPGs, adjusted for baseline characteristics (age, gender, tumor site, tumor size, histology in 12 subgroups and WHO grade, setting), categorical variables were included in univariate Cox proportional hazard regression models. In this study, expert center was identified as a structure seeing a high volume of sarcoma, with dedicated multidisciplinary sarcoma team and high level of molecular analysis, histological and radiologic second opinion activity.

Notably, because of a collinearity relationship between parameters defining adherence to CPGs, adherence was considered into the multivariate analysis using only the conformity to CPGs for the primary surgery procedures; indeed, this criterion showed the highest degree of significance in univariate analysis. Finally, independent prognostic variables of OS and PFS were respectively identified by multivariate Cox regression analysis, using a backward stepwise procedure to eliminate non-influential variables. We used a 0·10 significant level for entering and 0·05 for removing explanatory variables. All statistical analyses were performed with SAS v.9.3.

## Results

### Patient characteristics

All pathologists in the region were instructed to address all demonstrated or suspected cases of sarcoma for central review by the sarcoma pathologists’ team. Tumor characteristics, including site, localization, stage, histology and grade, are described in Table 1. The median time between the first medical visit and histological diagnosis was 23 days (95% CI: 29·9; 43·5). Patients and tumors characteristics were well balanced between expert and general hospitals.

**Table 1:** Patient characteristics and prognostic factors of progression-free survival in univariate analysis.

Patient characteristics		n = 472	Median PFS (months)	P value (log-rank test)
Sex			0·09	
Women	228 (48·3%)	NR *		
Men	244 (51·7%)	54·5		
Age (in years)			0·0001	
Median (min-max)		61 (16–92)		
≤ 60 years > 60 years			NR 40·9	
Grade^¥^			0·0001	
Low	203 (43.2%)	NR		
Intermediate	107 (22.8%)	43·9		
High	160 (34.0%)	16·5		
Localization			0·02	
Limbs		210 (44.5%)	59·0	
Other	262 (55.5%)	NR		
Size (in mm) ^†^			0·002	
Median (min-max) ≤ 50 mm > 50 mm		70 (5–400) 171 (38.4%) 274 (61.6%)	NR 50·1	
Setting^‡^			0·008	
Non-expert centers		293 (66·6%)	71·3	
Expert centers	147 (33·3%)	NR		
Main histological types			0·0001	
Angiosarcoma		17 (3.6%)	5·5	
Dermatofibrosarcoma protuberans	36 (7.6%)	NR		
Liposarcoma	133 (28.2%)	NR		
Leiomyosarcoma	69 (14.6%)	52·4		
Malignant peripheral nerve sheath tumor		11 (2.3%)	37·5	
Myxofibrosarcoma	21 (4.4%)	NR		
Soft tissue osteosarcoma	9 (1.9%)	11·3		
Rhabdomyosarcoma	10 (2.1%)	13·6		
Unclassified sarcoma	106 (22.5%)	26·5		
Synovial sarcoma	15 (3.2%)	NR		
Solitary fibrous tumor / hemangioendothelioma	10 (2.1%)	47·8		
Others^§^	35 (7.4%)	NR		
Adherence to CPGs for surgery ^£^			<0·0001	
Yes	249 (52·8%)	NR		
No	197 (41·7%)	45·2		

* NR: not reached,

^¥^ n = 470 (2 missing data),

^†^ n = 445 (27 missing data),

^‡^ n = 440 (32 missing data),

^§^ Epithelioid sarcoma, desmoplastic round-cell tumor, PEComa, intimal sarcoma, rhabdoid tumors, myxofibrosarcoma, inflammatory myofibroblastic tumors, myxoinflammatory fibroblastic sarcoma,

^£^ n = 446 (26 patients not evaluable)

### Adherence to CPGs

Overall, CPGs during diagnosis were conformed for only 277 patients of 469 (three cases with missing data), resulting in an adherence rate of 59·1% (95% CI: 54·5–63·5%). A multidisciplinary assessment by the Sarcoma Committee before surgery was conducted for 36·6% of patients treated in expert hospitals vs. 9.7% in general hospitals (p<0·001). A total of 440 patients (93·2%) underwent initial surgery, including 136 with negative histological margins (R0) (30·9%), 190 with macroscopically complete excision but positive margins (R1) (43·2%) and 114 with incomplete macroscopic excision (R2) (25·9%). Surgical procedures adhered to CPGs in 249 patients, corresponding to an adherence rate of 55·8% (95% CI: 51·1–60·5%). The most common reason for non-adherence to surgical procedures was the absence of planned surgery (n = 190). Regarding adjuvant therapy, chemotherapy and radiotherapy adhered to CPGs for 96% and 85% of patients respectively. The most common reason for non-adherence to chemotherapy and radiotherapy procedures were the non-used of RT or use of adjuvant CT for low grade and R0 or R1 surgeries Concerning follow up, 323 patients (84%) were monitored according to CPGs.

Taking into account all parameters, the overall adherence rate for the whole sequence (from diagnosis to post-treatment survey) was 36% (95% CI: 31·7–40·5%).

A significantly higher global adherence rate was observed in hospital expert in sarcoma management than in non-expert hospitals (57·1 vs. 19·5%; p<0.001).

### Local relapse and metastases

The median follow-up time from diagnosis was 62·7 months (ranging from 0 to 95·5). Tumor relapse was reported in 166 patients (35·2%), with a median time between diagnosis and first relapse of 11·5 months (ranging from 0·4 to 73·9). Local relapse was reported in 121 (25·6%) patients and metastatic recurrence in 101 (21·4%) patients. A total of 56 patients (11·9%) had both local and metastatic relapses.

### Progression-free survival

The median PFS of the 472 STS patients was 73·9 months (95% CI: 53·4-NR). Univariate analyses indicated that PFS was significantly associated with adherence to CPGs for surgery, age at diagnosis, tumor grade, tumor size, histology, tumor localization, as well as patient management in an expert hospital. There was also a trend towards a worse PFS for male patients (Table 1).

**Fig 2:**
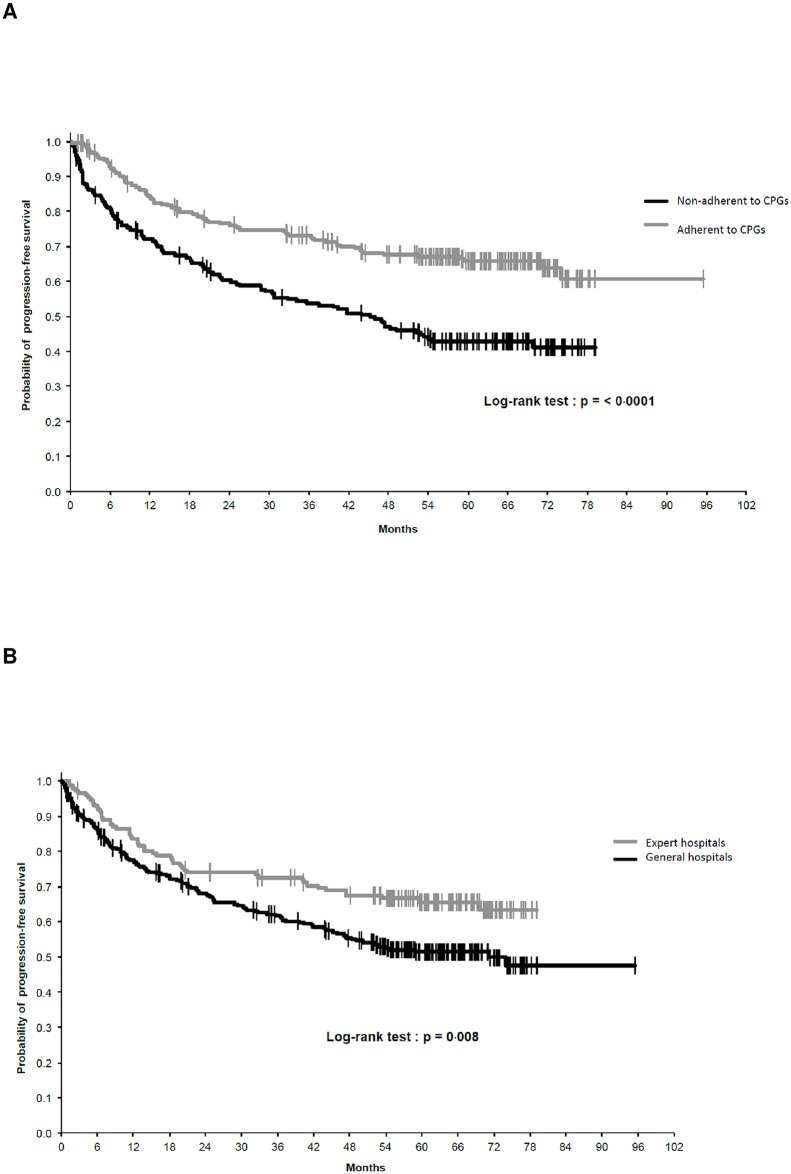
A: Progression-free survival and adherence to CPGs for surgery. B: Progression-free survival analysis according to expert centers.

Table 2 reports the multivariate analysis results of prognostic factors for PFS. The following independent favorable prognostic factors were identified: adherence to CPGs for surgery, young age (less than 60 years old) at diagnosis, female gender, WHO grade 1 or 2, tumor size ≤ 50 mm, and histology. Specifically for histology, angiosarcoma and soft tissue osteosarcoma were associated with a worse prognosis. Based on the HR, Adherence to CPGs for surgery as also shown in Fig 2A was one of the strongest independent predictor of PFS with histological grade. Fig 2B reported the progression free survival in expert centers versus PFS in non-expert centers.

**Table 2:** Progression-free survival for STS: multivariate analysis.

Factors	HR	95% CI	p-value
Adherence to CPGs for surgery	0·44	0·32–0·59	< 0·0001
Age in years at diagnosis (≤ 60 vs. > 60)	0·60	0·43–0·84	0·003
Sex (W vs. M)	0·70	0.52–0.95	0·02
Size (≤ 50 mm vs. > 50 mm)	0·61	0·43–0·86	0·005
Grade			<0·0001
Intermediate vs. High Low vs. High	0·64 0·20	0·42–0·96 0·12–0·32	
Histology (Liposarcoma are the reference)			0·0001
Angiosarcoma	4·67	2·13–10·21	
Dermatofibrosarcoma protuberans	0·44	0·10–1·92	
Leiomyosarcoma	1·02	0·61–1·73	
Malignant peripheral nerve sheath tumor	1·48	0·60–3·63	
Myxofibrosarcoma	0·42	0·17–1·05	
Soft tissue osteosarcoma	2·91	1·18–7·17	
Rhabdomyosarcoma	1·22	0·41–3·70	
Unclassified sarcoma	0·88	0·53–1·47	
Synovial sarcoma	0·63	0·23–1·74	
Solitary fibrous tumor + hemangioendothelioma	2·73	1·14–6·56	
Others	1·33	0·68–2·62	

A subset analysis was conducted using data from patients with liposarcoma, which was the most common histological subtype in this study. In multivariate analysis, surgical conformity significantly improved the PFS of patients with liposarcoma (HR: 0·32; 95% CI [0·16–0·61]), along with age at diagnosis ≤ 60 years (HR: 0·42; 95% CI [0·21–0·83]), grade (low vs. high, HR: 0·16, 95% CI [0·07–0·37]; intermediate vs. high, HR: 0·8, 95% CI [0·34–1·85]) and tumor site (limbs vs. abdominopelvic and retroperitoneal tumors HR: 0·49, 95% CI [0·24–1·01]; head, neck and thoracic vs. abdominal and retroperitoneal tumors HR: 0·12, 95% CI [0·02–0·93]).

### Overall survival

**Fig 3:**
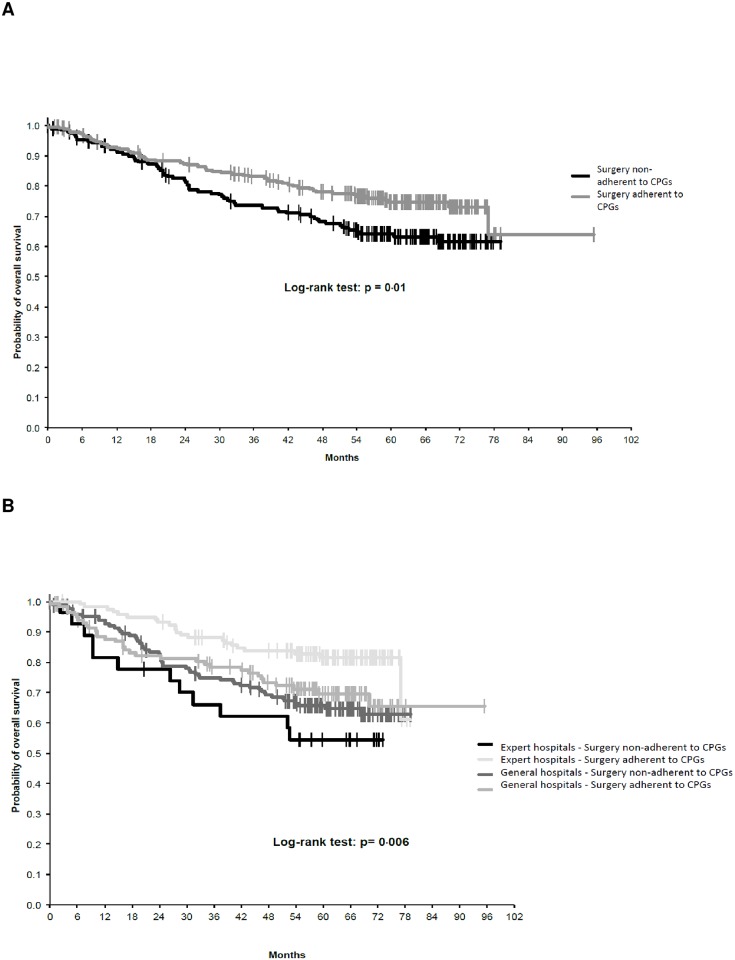
A: Overall survival: Overall survival and adherence to CPGs for surgery. B: Overall survival curves showing the interaction between adherence to CPGs for surgery and treatment centers.

Multivariate analyses revealed that the OS of STS patients was strongly influenced by adherence to CPGs for surgery (Fig 3A), with an interaction according to the organizational setting in which patients underwent surgery (Fig 3B). Stratification of OS by treatment center revealed a highly significant increase in OS for patients who underwent surgery in expert centers according to CPGs. In addition, age, tumor grade, histology and gender were independent prognostic factors for OS (Table 3). In the largest histological subset of patients with liposarcoma, adherence to CPGs for surgery was also an independent prognostic factor for OS.

**Table 3:** Overall survival for STS: multivariate analysis.

Factors	HR	95% CI	p-value
Interaction between Adherence to CPGs for surgery and setting (non-expert vs expert hospitals)			0.010
Adherence to CPGs for surgery in Non expert hospitals Adherence to CPGs for surgery in expert hospitals	- 0·34	- 0·16–0·68	0·99 0·003
Age in years (≤ 60 vs. > 60)	0·4	0·26–0·62	<0·001
Sex (W vs. M)	0·56	0·38–0·83	0·004
Grade			<0·0001
Intermediate vs. High Low vs. High	0·40 0·12	0·24–0·65 0·07–0·22	
Histology (Liposarcoma are the reference)			0·03
Angiosarcoma	2·54	1·15–5·63	
Dermatofibrosarcoma protuberans	0·36	0·05–2·71	
Leiomyosarcoma	0·85	0·46–1·59	
Malignant peripheral nerve sheath tumor	0·67	0·20–2·29	
Myxofibrosarcoma	0·40	0·15–1·08	
Soft tissue osteosarcoma	1·57	0·56–4·38	
Rhabdomyosarcoma	1·46	0·48–4·45	
Unclassified sarcoma	0·68	0·39–1·22	
Synovial sarcoma	0·56	0·16–1·93	
Solitary fibrous tumor + hemangioendothelioma	0·85	0·29–2·51	
Others	1·3	0·52–3·28	

## Discussion

Although several CPGs have been reported in the last 15 years, the initial management of STS patients often does not follow these recommendations in retrospective studies assessing their implementation [15–17]. This lack of adherence to guidelines has been suggested to be associated with an increased risk of relapse in several retrospective reports or single-center series. However, biases related to their retrospective nature and non-exhaustive collections of data have limited the conclusive values of these observations.

For these reasons, we initiated this population-based study exploring the determinants of outcome of an exhaustive series of patients from a well-defined geographical region, the RA region in France, which gathers 5.9 million inhabitants. All tumors were systematically reviewed by pathologist experts in sarcoma [8]. To our knowledge; this is the first research program to use this method in sarcoma. Because of its prospective exhaustive population-based nature, this series is not biased and, actually patient tumor localization and histology reflected those reported in international registries [22]. Based on such design, this is currently a highest level of evidence reported in the scientific literature, as randomized trials cannot be proposed to the patients to evaluate such impact on survival. The representativeness of the included population is also demonstrated by the other prognostic factors for PFS and OS identified here and were similar to those reported in other studies in patients with STS located in the extremities in large sarcoma registries [23,24].

With a median follow-up time of more than five years and taking into account all potential prognostic factors, we report that adherence to CPGs for surgery and treatment in an expert center for sarcoma are independent positive factors affecting PFS and OS in STS patients. If expert center management did not persisted in the multivariate analysis for PFS, is probably due to the highest conformity rate in the expert centers compared to non-expert centers. An extrapolation could be that conformity with CPG’s in expert centers is a confounding factor for PFS. Among patients who were treated in accordance to CPGs, OS was better in expert centers as compared to non-expert ones, with 66% reduction in the risk of death in patients with localized STS managed within expert centers.

In clinical trials, it has been shown that adherence to strictly defined guidelines during diagnosis and treatment affects clinical outcome and patient survival in highly selected patient populations [25]. However, although several studies have investigated the adherence to CPGs on disease management, few studies have assessed the correlation between adherence to CPGs and survival in oncology. Actually, data from patients with frequent cancers, such as breast cancer [26] or colon cancer [27], revealed a strong correlation between guideline-adherent therapeutic regimens and prolonged recurrence-free survival and OS. However, routine clinical procedures and implementation of CPGs is likely to be better established for frequent tumors than for more rare tumors such as STS. A recent study using the same methodology conducted on a sample of 151 sarcoma patients prospectively enrolled for two years in an Italian region (Veneto) within the frame of the European CONnective TIssue CAncer NETwork (CONTICANET), demonstrated that incomplete adherence to CPGs for the loco regional treatment of sarcomas was also associated with worse prognosis in patients with localized tumors [28].

The efficacy of the organization and management of sarcoma treatment has been the focus of several studies. Factors assessed in these studies included: delayed referral of patients to specialist centers; type of treatment center [14]; incomplete tumor resection; the existence of a sarcoma committee and centralization of the management [16,18]. These studies suggested (but did not demonstrated) that centralization was associated with a significant improvement in the clinical management of STS [13,15]. The present study confirms in a prospective & exhaustive population-based series that adherence to CPGs for surgery was higher in expert centers than in other institutions, and because these tumors tended to be larger, deep-seated and with highest histological grade, a recruitment bias is unlikely. The exact reason why expert centers adhered better to CPGs remains uncertain, but could be related to a consistent use of multidisciplinary treatment planning for these patients compared to other institutions. Interestingly, Adherence to CGP’s is a strongest prognostic factor for survival compared to surgical margins, as anticipated surgery also R1 or R2 seems better than a not so bad “whoops” surgery! Possibly, multidisciplinary assessment by the Sarcoma Committee before surgery, and the number of patients managed, improves overall experience of the team, time dedicated to anticipated management and therefore patient outcome, as already reported for other cancers [26,27]. In this exhaustive population based series, management within an expert center was associated with an improved PFS and OS. This observation represents a strong basis for the management of these patients in a small number of experience centers, as already implemented in several countries. It is noteworthy that there was an interaction between expert hospitals and quality of surgery for OS, indicating that the improvement of outcome in expert hospitals is not related only to the adherence to CPGs for surgery, but also to other aspects of patient management.

### How to improve adherence?

The global adherence rate was of 32% in a 1999–2000 study in patients with STS in the RA region [15] compared to 36% in the present study, therefore indicating a limited improvement in guideline adherence over this seven-year period. The low rate of adherence to CPGs in the present study shows that the publication of CPGs *per se* is insufficient to modify clinical practice. Methods for achieving widespread implementation of CPGs could include university education of medical students, radiologists training, lectures at local hospitals, sarcoma-focused education programs for general surgeons and radiologists, and the coordination of dedicated sarcoma treatment network. Currently available data, as well as this study, support the use of a centralized healthcare system for the management of sarcoma. Centralization of STS patients in expert centers, especially those with rare tumors, could minimize the risk of local recurrence and maximize survival time. The benefits obtained through centralization of patient management in expert centers could exceed the improvement in survival observed in all clinical trials performed in the context in these tumors in the last 20 years. Indeed, the magnitude of such improvement due to the adherence to CPGs for surgery (HR 0·44) should be balanced to that obtained with an adjuvant doxorubicin chemotherapy regimen currently proposed to patients with STS including similar population of patients where HR was only of 0·86. [29]

## Conclusion

This is the largest, most comprehensive dataset reported to date on the adherence to CPGs in an exhaustive population of patients with localized STS confirmed by centralized pathology. Adherence to CPGs was low and adherence to surgical guidelines was found to be a key predictor of survival with a reduction of 66% in the risk of death among patients treated in reference centers. These data highlight and quantify clinically significant disparities in the quality of sarcoma care and OS, demonstrating for the first time that patients referred to an expert center for primary resection were more likely to be managed according to CPGs than patients treated in general hospitals. Increased efforts are needed to improve the implementation of CPGs, in particular before and during surgery.

## Supporting information

S1 Appendix List of pathologists of the Rhone-Alpes region who actively collaborated in the study. (DOC)

S2 Appendix List of pathologists of the Rhone-Alpes region who actively collaborated in the study. (DOC)
