# Supplementary material for: Survival impact of centralization and clinical guidelines for soft tissue sarcoma (A prospective and exhaustive population-based cohort)
Source: PLoS One. 2017 Feb 3;12(2):e0158406. doi: 10.1371/journal.pone.0158406 (PMC5291382; doi:10.1371/journal.pone.0158406)
Supplement: S1 Appendix — (DOC) [file pone.0158406.s001.doc]

**List of pathologists of the Rhone-Alpes region who actively collaborated in the study**

AGARD C, ALLIAS-MONTMAYEUR F, ANGONIN R, AUGROS-MONAVON M, BAILLY C, BALME B, BANCEL B, BARNOUD R, BEN-LAGHA N, BENSAADI L, BERGER F, BERGER DUTRIEUX N, BESCHET I, BILLARD F, BLANC V, BONIN AM, BOTTERO N, BOURLOUX J, BOUTONNAT J, BOUVIER R, BOZON C, BRAMBILLA E, BRINGEON B, BUENEMD A, BUYCK-MABRUT M, CANTERO B, CAVAILLE CS, CHALABREYSSE L, CHALABREYSSE P, CHAMBONNIERE ML, CHANOZ J, CHANOZ-POULARD G, CHASSAGNE-CLEMENT C, CHEVALIER M, CHOUVET B, CIAPA A, CLARET-TOURNER C, CLEMENSON A, COLLARDEAU FRACHON S, CORRAND-FAURE A, CORSOIS L, CROZES F, CRUEL I, DARDELIN R, DAVID C, DECAUSSIN M, DECOUVELAERE AV, DE LA FOUCHARDIERE A, DENIER JF, DEPARDON-DOLCE J, DER GARABEDIAN P, DEROLLAND P, DESCOMBES-THIVOLET B, DEVOUASSOUX M, DIENY A, DIJOUD F, DONNE C, DONSBECK AV, DONZEL JP, DOUCHET C, DUMOLLARD JM, ECONOMIDES A, ELBAZ N, FABRE B, FAISANT M, FAURE C, FAYSSE M, FELMAN P, FEUTRY C, FRENCH M, FIOR-GOLZAN M, FRAPPART L, GASNIER F, GENTIL-PERRET A, GLEHEN A, GODARD W, GODENECHE J, GOUARDERES C, GOUZY-GROSJEAN F, GRIOT A, GUILLAUBEY C, GUILLAU C, HERVE-NICOLLET C, HERVIEU V, ISAAC-PINET S, ISTIER L, JOUFFRE-COTTIER M, JOUVET A, KANITAKIS J, KERMANAC’H P, KHADDAGE A, KNOPF JF, KOEB MH, LABADIE M, LAMOULIATTE B, LANTUEJOUL S, LAURENT I, LAURO-COLLEAUX C, LAVERRIERE MH, LE BRETON F, LE MARC’HADOUR F, LUCHT-VERSINI P, MAC GREGOR B, MACHAYEKHI JP, MAISONNEUVE-GILLY D, MARTIN H, MEGE LECHEVALLIER F, MESGUICH P, MEYRONET D, MORAND-DUSSERRE I, MORCILLO JL, MOREL F, MORENS A, MULLER B, MULLER C, NEY M, NEYRA M, PASQUIER B, PASQUIER D, PAULIN C, PEOC’H M, PERROT G, PIALAT J, PIATON E, PICCHETTI MAYER N, PINET BRIQUET N, PLENIER MAISONNEUVE M, POCACHARD P, PUGENS G, REMY I, RANCHERE-VINCE D, RICHARD J, ROUAULT-PLANTAZ V, ROUX JJ, ROUX-GILLY MG, SAINT-GENIS L, SAINT-PIERRE G, SALAMEIRE D, SALLE M, SALON C, SCOAZEC JY, SEIGNEURIN D, SERAIN F, SICHE L, SOUBEYRAND MS, STREICHENBERGER N, STURM N, SUIGNARD Y, TERDJMAN P, THIVOLET-BEJUI F, TREILLEUX I, VANCINA S, VAUNOIS B, VERCHERIN A, VITTETAT F, VITREY D, VOCK-BONNET M, ZAPPATINI L
