# Supplementary material for: Survival impact of centralization and clinical guidelines for soft tissue sarcoma (A prospective and exhaustive population-based cohort)
Source: PLoS One. 2017 Feb 3;12(2):e0158406. doi: 10.1371/journal.pone.0158406 (PMC5291382; doi:10.1371/journal.pone.0158406)
Supplement: S2 Appendix — (DOC) [file pone.0158406.s002.doc]

**List of physicians who collaborated in the study**

ABELLARD J, ACHOUR Y, ADHAM M, AFCHAIN JM, AL NAASAN I, ALACOQUE B, ALESSIO A, ALLAGNAT B, ALLAMEL G, ALLEGRE JP, AMOYAL P, AMROUN H, ANGELLA S, ANTOINE P, ARDISSON P, ARIMONT JM, ARLAUD R, ARNAUD-CROZAT E, ARNOULD H, ARPIN N, ARVIEUX C, ASSOULINE D, AUBERT A, AUBERT M, AULAGNER G, AVRIL G, AZOULAI JJ, BACCOT S, BACLE B, BAGEACU S, BAHUREL J, BALIQUE JG, BARBE C, BARBIER B, BARLETTA H, BARRIER PY, BAUCHU P, BAULIEUX J, BEATRIX O, BEAUDOUIN E, BEAUNE J, BEAUNE B, BEGUIN L, BELGADI B, BELLIARD H, BELLON-CHAMPEL P, BENCHETRIT S, BENJELLOUN A, BERARD P, BERARD J, BERGER E, BERGER C, BERGER JL, BERLIER JL, BERTHEAS M, BETHMONT T, BEZIAT JL, BIGAY D, BILLIET C, BLACHE G, BLONDET R, BOACHON A, BOBICHON R, BOIBIEUX A, BOILLOT I, BOILLOT O, BOLZINGER E, BONJEAN JA, BONNARD O, BOREL I, BOTTERO L, BOUARIOUA N, BOUCHARD F, BOUCHET B, BOUCHET P, BOUDIAF A, BOUHOUR D, BOUILLON P, BOULET S, BOULETREAU P, BOULEZ JC, BOURBON M, BOURDARIAT R, BOURDEIX O, BOURELLE S, BRAUD G, BRETON C, BRETON P, BRICHON PY, BRINGEON G, BRIX M, BRUN O, BRUNON J, BRUTZKUS A, BUISSON L, BURGARD G, CABAUD V, CAILLON P, CAILLOT JL, CALLOC'H F, CAQUANT L, CARRET JP, CASSIGNOL A, CATIMEL G, CATTEY JAVOUHEY AC, CAVARD I, CERUSE P, CHABAUD B, CHADOINT F, CHALBET JY, CHAMBAUD D, CHAMBON M, CHAMPETIER T, CHAMPION F, CHAPELLE S, CHARLES JP, CHASSAGNE S, CHASSARD M, CHATELARD P, CHATELET JC, CHAULEUR C, CHAVATTE PY, CHEFAI M, CHEKAROUA K, CHENET P, CHEVALLIER C, CHIRPAZ A, CHOL R, CHOLIN N, CHOTEL F, CHRISTOPHE M, CLAUDY A, CLAVERANNE B, CLIPPE C, COCCHI P, COEFFIC D, COGNAT T, COLLARD O, COLLEAUX B, COMBEMALE P, COMMUNAL PH, COMTE CE, CORCELLA D, CORDIER JF, CORRONT B, COTTALORDA M, COURTOIS C, CROCHET J, CUCHET E, CZYGLIK O, DALLE S, DANAN M, DAUDE O, DE LA ROCHE E, DE MARLIAVE H, DE MOURGUES P, DE PAEPE JP, DE SAINT HILAIRE P, DE VILLENEUVE L, DEBBAGH B, DEBOURDEAU P, DEJOUR D, DELANNOY P, DELAPORTE T, DELBAERE DELBECQUE M, DENIS E, DESCOURS H, DESMETTRE O, DESROCHES E, DESSENON C, DESTRUMELLE N, DIELEMAN P, DILIN C, DILOU N, D'INGRADO P, DISSARD C, DJOUHRI F, DOJCINOVIC S, DOLMAZON C, DONNE R, DUBOIS R, DUCERF C, DUCROUX A, DUJARDIN P, DUMAS B, DUMONT P, DUPRE LA TOUR L, DUPREZ D, DURAND JM, DURAND PY, DUTHEL R, DUTOIT M, DUYCK JP, DYON JF, EID A, EVEN P, EYMARD P, EYRAUD G, FARIZON F, FAUCOMPRET S, FAURE C, FAURE JL, FAVRE JP, FAYAD S, FAYETTE J, FAYSSE E, FELLONI B, FERRIER C, FERRIERE C, FESSY MH, FINET P, FIQUET A, FONTAUMARD E, FOREST G, FOREST G, FORLI A, FOTSO MJ, FOUILLARD C, FOURNIER B, FRANCOIS Y, FRASSINETTI E, FRERING V, FRESSINET RM, FREY Gil FROBERT JL, FROEHLICH P, FROISSART B, GAMONDES JP, GARBIT V, GARRET J, GAUTHIER G, GERBAUD B, GLEHEN O, GLEIZE B, GLOPPE H, GODENECHE A, GOLFIER F, GONNET E, GOUILLON C, GRANDCLEMENT E, GRANDJEAN JP, GRANGER P, GRESTA G, GRIOT JP, GUIBERT B, GUILLEM P, GUYOTAT J, HABOZIT B, HAMADE A, HAMEURY F, HENRY C, HERITIER P, HERZBERG G, HOCH M, HOKAYEM D, HUGONNIER G, HUPPERT J, ISOARD L, JABOT VIGNEAUD H, JACQUEMIER D, JACQUOT F, JANODY P, JARSAILLON P, JEGOU C, KACZMAREK D, KEPENEKIAN G, KHALAF M, KIRCHMEIER M, KOHLER R, KOYAZOUNDA A, KRAFT FX, KRIFI S, KUDELA I, LABOREY BROUILLOUX C, LABROSSE H, LACROIX B, LAGOUTTE J, LAMBLIN G, LANGERON D, LARAMAS M, LATIL JL, LATUNE D, LE DERF Y, LE GENISSEL H, LE MEUR P, LECCIA MT, LEDOYEN C, LESCURE G, LETOUBLON C, LEVAL J, LIENHART J, LIFANTE C, LILLE R, LIMONNE B, LITAS P, LITOR M, LOUIS C, LOURY JN, LUCIANI RC, MABRUT JY, MACHENAUD A, MAHLA K, MAIRE J, MAISONNETTE F, MAITREJEAN Y, MALERE G, MALLECOURT P, MAMMAR V, MARCHAND B, MARCHAT F, MARECHAL E, MARGOTTON J, MARGOTTON A, MARTINET X, MARTINEZ T, MASSART P, MATHEVET P, MAYER B, MEEUS P, MEHDI A, MELERE G, MENOUILLARD O, MERLOZ P, MERMET J, MESTRALLET JP, METOIS P, MEUNIER A, MEUNIER P, MEYER JY, MICHEL A, MIGNOTTE H, MILOU F, MINGUET C, MITHIEUX F, MONSALIER M, MOREL JJ, MOTTOLESE C, MULLER P, MULSANT P, MURE PY, NAPOLEON B, NASSAR E, NAVAILLES B, NAWABI H, NESSAH BOUSQUET K, NICOLINI P, NIKOLITCH G, NOGIER M, NOURI K, NOURRISSAT C, OLAGNE E, OUILLON-VILLET C, OULIE O, OUSSOFF B, PADET JM, PAPAREL P, PAPILLON M, PAPIN P, PARIS D, PARTENSKY C, PATET JD, PAULE R, PEGAZ FIORNET M, PEGORIER O, PEIX JL, PELISSIER F, PERRIN P, PERROT F, PETIOT ROLAND A, PETIT L, PEYRAT P, PIC JC, PICARD JL, PIGNAT JC, PIOLAT C, PLAWECKI S, POISSON JF, POULARD V, PRADEL P, PRADES JM, PROST P, PROVENSAL BERTRAND AM, PUGEAT G, RACHIDI A, RASATA J, RAUDRANT D, RAYMOND A, REGAIRAZ C, RENY P, REY A, REY Y, REY JC, REYT E, RIGAUD P, RIGHINI C, RISSE O, RIVIER R, RIVOIRE M, RKIBA M, ROBART S, ROBIN B, ROCHE M, ROGET L, ROLET JP, ROMESTAING P, RONDELET O, ROSSI J, ROSTOUCHER P, ROUX JF, ROUX O, RUBAN JM, RUIZ JM, SAUTIER M, SCHMITT T, SCHREIBER V, SCOWRON O, SEFFERT P, SEGUIN P, SENELLART F, SERVAJEAN V, SESSA C, SEULIN P, SEUTIN B, SINDOU M, SKOWRON F, SORIANO E, SOUALMI M, SOUCHERE B, SPITALIER P, STEFANI L, STREICHENBERGER T, SUCHAUD JP, TALABARD JN, TARDIF D, TAVAN D, TAVIN B, THOMAS D, THOMAS L, TIFFET O, TOHOUBI W, TONETTI J, TOSTAIN J, TRONC F, VAITON JC, VALLA V, VALLEE B, VANEL O, VARGAS BARETTO B, VARLET F, VAUDAINE JP, VAZ G, VERNAY L, VERNAY A, VEYRE DE SORAS X, VIAL P, VIGNEAU B, VILLARD P, VINCENT B, VOCHE P, VOILIN C, VOIRIN D, VOLLE F, VOUREY G, WAUTOT F, YOU JE, YOUVARLAKIS P, YVER R, ZARKA A, ZENOU E, ZROUNBA P
